# Supplementary material for: Bmal1 in the striatum influences alcohol intake in a sexually dimorphic manner
Source: Commun Biol. 2021 Oct 26;4:1227. doi: 10.1038/s42003-021-02715-9 (PMC8548330; doi:10.1038/s42003-021-02715-9)
Supplement: Supplementary file 2 — Supplementary Information [file 42003_2021_2715_MOESM2_ESM.pdf]

Bmal1 in the striatum influences alcohol intake in a sexually dimorphic manner

Nuria de Zavalia*, Konrad Schoettner, Jory A. Goldsmith, Pavel Solis, Sarah Ferraro, Gabrielle Parent, and Shimon Amir*

Center for Studies in Behavioral Neurobiology

Department of Psychology

Concordia University

Montreal

Canada

* Correspondence: shimon.amir@concordia.ca, nuria.dezavalia@concordia.ca

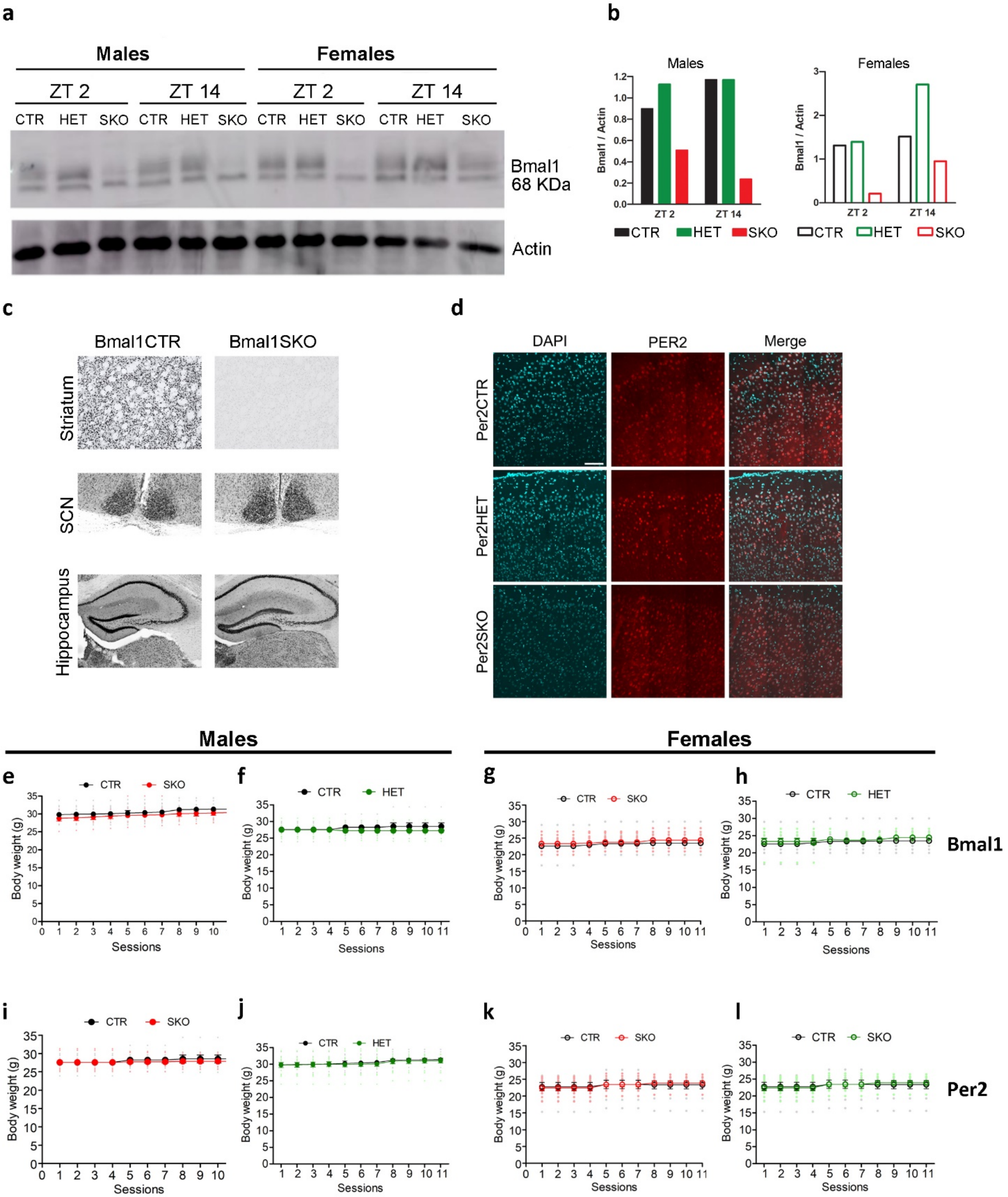
a
Males
Females
ZT 2
ZT 14
ZT 2
ZT 14
CTR HET SKO CTR HET SKO CTR HET SKO CTR HET SKO
Bmal1
68 KDa
Actin
b
Males
Females
Bmal1 / Actin
ZT 2
ZT 14
CTR HET SKO
CTR HET SKO
c
Bmal1CTR
Bmal1SKO
Striatum
SCN
Hippocampus
d
DAPI
PER2
Merge
Per2CTR
Per2HET
Per2SKO
Males
Females
e
f
g
h
CTR
SKO
HET
Body weight (g)
Sessions
Bmal1
i
j
k
l
Per2

Supplementary Figure 1: *Bmal1* expression in the mouse striatum and body weight of *Bmal1* and *Per2* knockout mice.

a, Qualitative western blot analysis of BMAL1 in the dorsal striatum of control and *Bmal1* knockout male and female mice at two different times of the day. **b**, Quantitication of the blot intensity is shown. **c**, a representative image of BMAL1 immunohistochemistry staining in the striatum, hippocampus and SCN of control and Bmal1 knockout. **d**, a representative image of PER2 immunofluorescence staining in the cortex of control, *Per2* heterozygote and knockout mice. PER2: red, DAPI: cyan. Scale bar = 100 μm. **e**, daily body weight of control and *Bmal1* knockout male mice. Two-way repeated measure ANOVA (RM-ANOVA), no significant effect, F= (1, 23) = 0.9306, p=0.3448. **f**,daily body weight of control and *Bmal1* heterozygote male mice. RM-ANOVA, no significant effect, F (1, 26) = 0.05852, p=0.8108. **g**, daily body weight of control and *Bmal1* knockout female mice. RM-ANOVA, no significant effect, F (1, 29) = 0.8145, p=0.3742. **h**, daily body weight of control and *Bmal1* heterozygote female mice. RM-ANOVA, no significant effect, F (1, 25) = 0.6254, p=0.4365. **i**, daily body weight of control and *Per2* knockout male mice. RM-ANOVA, no significant effect, F= (1, 14) = 0.9905, p=0.7576. **j**, daily body weight of control and *Per2* heterozygote male mice. RM-ANOVA, no significant effect, F (1, 13) = 0.4200, p = 0.5282. **k**, daily body weight of control and *Per2* knockout female mice. RM-ANOVA, no significant effect, F (1, 17) 0.02185, p= 0.9633. **l**, daily body weight of control and *Per2* heterozygote female mice. RM-ANOVA, no significant effect, F (1, 13) = 0.2798, p= 0.6058.ZT: Zeitgeber time. **e-l**, the values express mean ± S.E.M. **a-d**, n= 3/genotype. **e-h**, CTR: control, HET: *Bmal1* heterozygote, SKO: *Bmal1* knockout. **e, f**, CTR n = 12, HET n = 16, SKO n = 13. **g, h**, CTR n = 17, HET n = 10, SKO n = 14. **i-l**, CTR: control, HET: *Per2* heterozygote, SKO: *Per2* knockout. **i, j**, CTR n = 8, HET n = 7, SKO n = 8. **k,l**, CTR n = 9, HET n = 6, SKO n = 10.

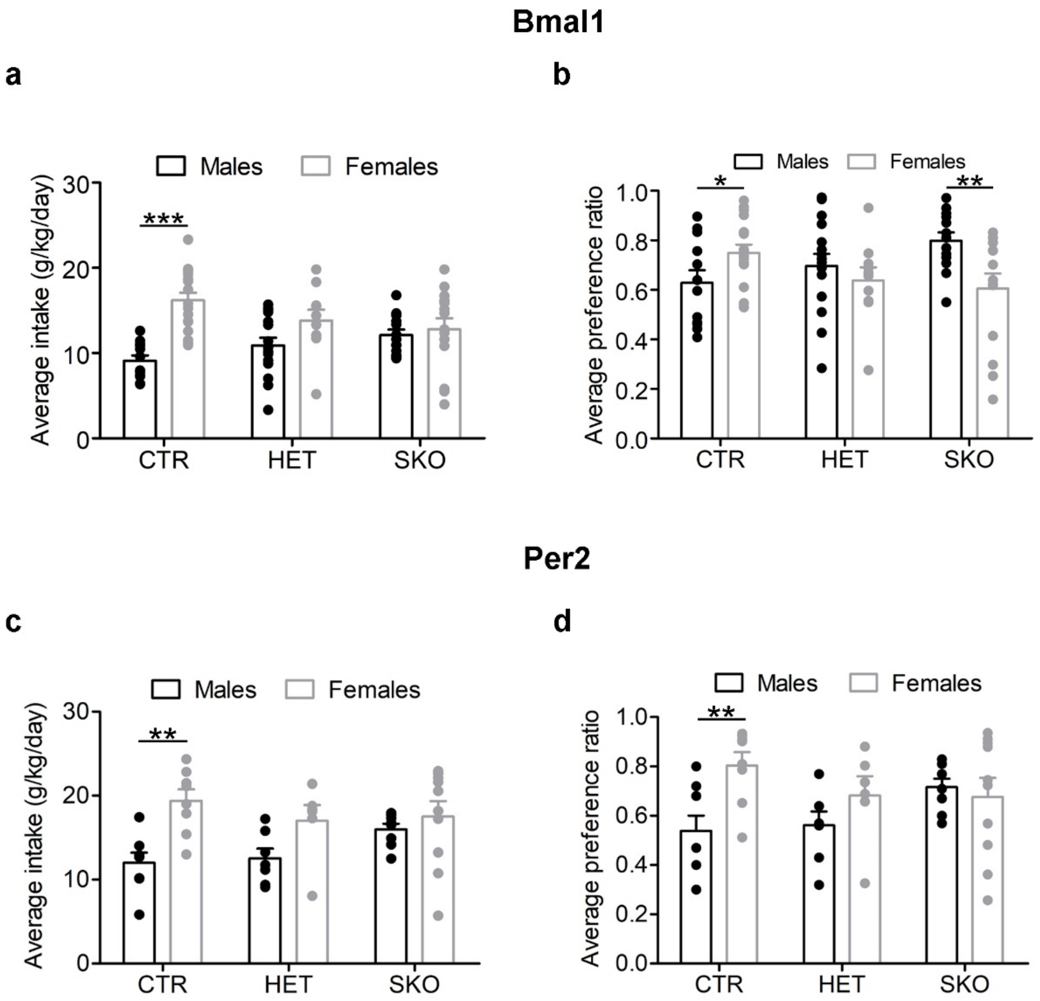
Bmal1
a
b
Males
Females
Average intake (g/kg/day)
30
20
10
0
***
CTR
HET
SKO
Average preference ratio
1.0
0.8
0.6
0.4
0.2
0.0
*
**
Per2
c
d
**
**

Supplementary Figure 2: Deletion of *Bmal1* eliminates sex differences in alcohol drinking behavior. **a**, average alcohol intake of male vs. female Bmal1 control, heterozygote and knockout mice. Unpaired two-tailed t-test, CTR male vs female *** p< 0.0001, HET male vs female, NS, p= 0.06, SKO male vs female, NS, p= 0.65. **b**, average alcohol preference of male vs. female Bmal1 control, heterozygote and knockout mice. Unpaired two-tailed t-test, CTR male vs female * p< 0.05, HET male vs female, NS, p= 0.36, SKO male vs female, ** p< 0.01. **c**, average alcohol intake of male vs. female Per2 control, heterozygote and knockout mice. Unpaired two-tailed t-test, CTR male vs female ** p< 0.01, HET male vs female, NS, p= 0.06, SKO male vs female, NS, p= 0.49. **d**, average alcohol preference of male vs. female Per2 control, heterozygote and knockout mice. Unpaired two-tailed t-test, CTR male vs female ** p< 0.01, HET male vs female, NS, p= 0.22, SKO male vs female, NS, p= 0.67.

NS = no significant differences. The value express mean ± S.E.M. **a, c**, CTR: Bmal1 control, HET: Bmal1 heterozygote, SKO: Bmal1 Knockout. Males: CTR n = 12, HET n = 16, SKO n = 13. Females: CTR n = 17, HET n = 10, SKO n = 14. **b,-d**, CTR: Per2 control, HET: Per2 heterozygote, 2SKO: Per2 Knockout. Males: CTR n = 8, HET n = 7 SKO n =8. Females: CTR n = 9, HET n = 6, SKO n = 10.

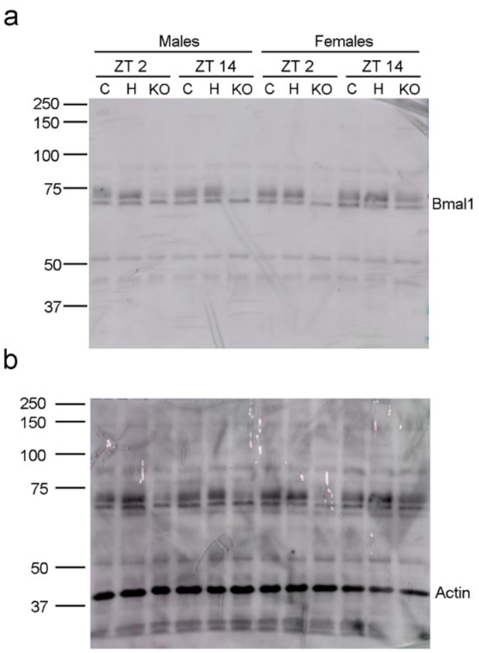

Supplementary Figure 3: Non-cropped Western blot gel from Supplementary Figure 1a.

a, Non-cropped anti-Bmal1 immunoblotting for BMAL1 in Supplementary Figure 1a. **b**, anti-Actin immunoblotting for ACTIN in Supplementary Figure 1a.
