# Supplementary material for: Bmal1 in the striatum influences alcohol intake in a sexually dimorphic manner
Source: Commun Biol. 2021 Oct 26;4:1227. doi: 10.1038/s42003-021-02715-9 (PMC8548330; doi:10.1038/s42003-021-02715-9)
Supplement: Supplementary file 3 — Description of Additional Supplementary Files [file 42003_2021_2715_MOESM3_ESM.pdf]

Description of Additional Supplementary Files

File name: Supplementary Data 1.
Description: Voluntary alcohol consumption in *Bmal1* knockout mice.

File name: Supplementary Data 2.
Description: Voluntary alcohol consumption in *Per2* knockout mice.

File name: Supplementary Data 3.
Description: Voluntary alcohol consumption in *Bmal1* and *Per2* heterozygote mice.

File name: Supplementary Data 4.
Description: Sucrose consumption in *Bmal1* and *Per2* knockout mice.

File name: Supplementary Data 5.
Description: Circadian analysis of locomotor activity of *Bmal1* and *Per2* control, heterozygote and knockout male and female mice.

File name: Supplementary Data 6.
Description: Voluntary alcohol consumption in *Gpr88* heterozygote mice.
