# Supplementary material for: Bmal1 in the striatum influences alcohol intake in a sexually dimorphic manner
Source: Commun Biol. 2021 Oct 26;4:1227. doi: 10.1038/s42003-021-02715-9 (PMC8548330; doi:10.1038/s42003-021-02715-9)
Supplement: Supplementary file 10 — Reporting Summary [file 42003_2021_2715_MOESM10_ESM.pdf]

nature research

Corresponding author(s): Shimon Amir
Nuria de Zavalia

Last updated by author(s): Aug 30, 2021

Reporting Summary

Nature Research wishes to improve the reproducibility of the work that we publish. This form provides structure for consistency and transparency in reporting. For further information on Nature Research policies, see our Editorial Policies and the Editorial Policy Checklist.

Statistics

For all statistical analyses, confirm that the following items are present in the figure legend, table legend, main text, or Methods section.

n/a	Confirmed	
☐	☒	The exact sample size (*n*) for each experimental group/condition, given as a discrete number and unit of measurement
☐	☒	A statement on whether measurements were taken from distinct samples or whether the same sample was measured repeatedly
☐	☒	The statistical test(s) used AND whether they are one- or two-sided<br>*Only common tests should be described solely by name; describe more complex techniques in the Methods section.*
☐	☐	A description of all covariates tested
☐	☒	A description of any assumptions or corrections, such as tests of normality and adjustment for multiple comparisons
☐	☒	A full description of the statistical parameters including central tendency (e.g. means) or other basic estimates (e.g. regression coefficient) AND variation (e.g. standard deviation) or associated estimates of uncertainty (e.g. confidence intervals)
☒	☐	For null hypothesis testing, the test statistic (e.g. *F*, *t*, *r*) with confidence intervals, effect sizes, degrees of freedom and *P* value noted<br>*Give P values as exact values whenever suitable.*
☒	☐	For Bayesian analysis, information on the choice of priors and Markov chain Monte Carlo settings
☒	☐	For hierarchical and complex designs, identification of the appropriate level for tests and full reporting of outcomes
☒	☐	Estimates of effect sizes (e.g. Cohen's *d*, Pearson's *r*), indicating how they were calculated

Our web collection on statistics for biologists contains articles on many of the points above.

Software and code

Policy information about availability of computer code

Data collection	Data were acquired with commercially available softwares indicated in the Methods section: Vitalview, Clocklab, Graphpad Prism, ImageJ and Fiji Plugin.
Data analysis	Data were analyzed with commercially available softwares indicated in the Methods section: Vitalview, Clocklab, Graphpad Prism, ImageJ and Fiji Plugin.

For manuscripts utilizing custom algorithms or software that are central to the research but not yet described in published literature, software must be made available to editors and reviewers. We strongly encourage code deposition in a community repository (e.g. GitHub). See the Nature Research guidelines for submitting code & software for further information.

Data

Policy information about availability of data

All manuscripts must include a data availability statement. This statement should provide the following information, where applicable:

- Accession codes, unique identifiers, or web links for publicly available datasets
- A list of figures that have associated raw data
- A description of any restrictions on data availability

The data sets generated and/or analysed during the current study are available from the corresponding author on reasonable request.

Field-specific reporting

Please select the one below that is the best fit for your research. If you are not sure, read the appropriate sections before making your selection.

☒ Life sciences ☐ Behavioural & social sciences ☐ Ecological, evolutionary & environmental sciences

For a reference copy of the document with all sections, see nature.com/documents/nr-reporting-summary-flat.pdf

Life sciences study design

All studies must disclose on these points even when the disclosure is negative.

Sample size	The number of animals required for the proposed experiments was calculated based on literature and previous experiments done in our lab.
Data exclusions	We did not exclude any data from analysis.
Replication	All attempts at replication were successful.
Randomization	Data collection and analysis were not performed randomized.
Blinding	Data collection and analysis were not performed blinded.

Reporting for specific materials, systems and methods

We require information from authors about some types of materials, experimental systems and methods used in many studies. Here, indicate whether each material, system or method listed is relevant to your study. If you are not sure if a list item applies to your research, read the appropriate section before selecting a response.

Materials & experimental systems

n/a	Involved in the study	
☐	☒	Antibodies
☒	☐	Eukaryotic cell lines
☒	☐	Palaeontology and archaeology
☐	☒	Animals and other organisms
☒	☐	Human research participants
☒	☐	Clinical data
☒	☐	Dual use research of concern

Methods

n/a	Involved in the study	
☒	☐	ChIP-seq
☒	☐	Flow cytometry
☒	☐	MRI-based neuroimaging

Antibodies

Antibodies used	Bmal1 rabbit polyclonal antibody, 1:1000 and 1:10,000 dilutions, Novus Biologicals, # NB100-2288, Littleton, CO, USA. Goat anti-rabbit IgG horseradish peroxidase-conjugated antibody, 1:200 dilution; Millipore Sigma, # AP132P, Burlington, MA, USA. Biotinylated anti-rabbit IgG, raised in goat, 1:200, Vector Laboratories, Burlington, ON, Canada. PER2 rabbit polyclonal antibody, 1:500, Novus Biologicals, # NB300-125, Littleton, CO, USA. Anti-rabbit secondary Alexa-647, 1:500, Life Technologies, Carlsbad, CA, USA.
Validation	All antibodies were validated by the manufacturer or publications.<br>mal1 rabbit polyclonal antibody: Perelis M, Marcheva B, Ramsey KM et al. Pancreatic B cell enhancers regulate rhythmic transcription of genes controlling insulin secretion. Science. 2015 Nov 06 [PMID: 26542580].<br>Izumo M, Pejchal M, Schook AC et al. Differential effects of light and feeding on circadian organization of peripheral clocks in a forebrain Bmal1 mutant Elife. 2014 Dec 18 [PMID: 25525750].<br>PER2 rabbit polyclonal antibody: Zhang XY, Wang L, Yan WJ et al. Period 2-Induced Activation of Autophagy Improves Cardiac Remodeling After Myocardial Infarction Hum. Gene Ther. Dec 10 2019 [PMID: 31822134].<br>Dong E, Guidotti A, Zhang H et al. Prenatal stress leads to chromatin and synaptic remodeling and excessive alcohol intake comorbid with anxiety-like behaviors in adult offspring Neuropharmacology Sep 15 2018 [PMID: 30016666].

Animals and other organisms

Policy information about studies involving animals; ARRIVE guidelines recommended for reporting animal research

Laboratory animals	Conditional knockout mice lacking BMAL1 or PER2 protein in the striatum were generated by two genetic crosses. In a first cross, Gpr88(Cre/+) male mice (B6.129S4-Gpr88tm1.1(cre/GFP)Rpa/J; stock number 022510; Jackson Laboratory) were bred with Bmal1(fl/fl) (B6.129S4(Cg)-Arntltm1Weit/J; stock number 007668; Jackson Laboratory) or Per2(fl/fl) (B6.129-Per2tm1.2Ual/Biat, strain ID: EM10599, European Mouse Mutant Archive) female mice to generate respective heterozygote F1 progeny ([Gpr88Cre/+; Bmal1fl/+]

or [Gpr88Cre/+; Per2fl/+]). In a second step, F1 males were crossed with Bmal1(fl/fl) or Per2(fl/fl) females to generate desired experimental and control animals. All floxed and Cre-expressing transgenic mouse lines have been backcrossed onto a C57BL/6J background for at least 6 generations. Conditional Bmal1 and Per2 knockout mice (Gpr88Cre/+; Bmal1fl/fl [Bmal1SKO], Gpr88Cre/+; Per2fl/fl [Per2SKO]), littermate heterozygote (Gpr88Cre/+; Bmal1fl/+ [Bmal1HET], Gpr88Cre/+; Per2fl/+ [Per2HET]) and wild type control animals (Gpr88+/+; Bmal1fl/fl [Bmal1CTR], Gpr88+/+; Per2fl/fl [Per2CTR]) of both sexes as well as Gpr88Cre/+ and corresponding control male and female mice were used for experiments with an age of 12-18 weeks.

Wild animals	This study did not involve the use of wild animals
Field-collected samples	This study did not involve the use of field collected samples
Ethics oversight	All procedures were approved by the Animal Care Committee of Concordia University, and performed in accordance with the institutional and the Canadian Council of Animal Care guidelines.

Note that full information on the approval of the study protocol must also be provided in the manuscript.
